# Highly Strong and Transparent Hydrogel Elastomers Microfabricated for 3D Microphysiological Systems

**DOI:** 10.1021/acsami.5c07880

**Published:** 2025-07-10

**Authors:** Wenxiu Li, Lianxin Li, Huimin He, Wang Peng, Zhengdong Zhou, Wanqing Wu, Dong Lv, Yaqing Chen, Wending Pan, Xiaoyu Zhou, Jun Yin, Mengsu Yang

**Affiliations:** † Institute for Frontier Science, 47854 Nanjing University of Aeronautics and Astronautics, Nanjing 210016, P. R. China; ‡ Department of Biomedical Sciences, and Tung Biomedical Sciences Centre, 53025 City University of Hong Kong, Hong Kong SAR 999077, P. R. China; § Department of Precision Diagnostic and Therapeutic Technology, City University of Hong Kong Matter Science Research Institute (Futian), Shenzhen 518057, P. R. China; ∥ Department of Mechanical Engineering, 25809 The University of Hong Kong, Hong Kong SAR 999077, P. R. China

**Keywords:** poly(vinyl alcohol) hydrogel, mechanical robustness, transparency, microfabrication, microphysiological systems

## Abstract

3D microarchitected hydrogels have recently been exploited to establish microphysiological systems for preclinical studies. However, promising hydrogels, unlike anhydrous elastomers, which have been widely adopted for device microfabrication, are still scarce for biodevice engineering due to their limitations in mechanical properties and manufacturability. Here, we leverage temperature-controlled physical cross-linking of a polymer network to generate highly strong, elastic, and transparent hydrogels, which can be further readily microfabricated into elaborate constructs for diverse device designs. Specifically, with the addition of a good solvent of dimethyl sulfoxide, poly­(vinyl alcohol) dissolved in the mixed solvent of dimethyl sulfoxide/water (4:1) shows extensive physical cross-links of nanosized polymeric crystallites upon one single freeze–thaw cycle, leading to the resulting hydrogels (∼80% water content) with superior mechanical properties and optical transparency, comparable to or even exceeding the anhydrous elastomer of polydimethylsiloxane. Furthermore, the simple processing technologies enable the patterning of hydrogels (high resolution of 20 μm) customized for various in vitro models, as exemplified by hydrogel microwell arrays supporting efficient tumor-spheroid generation and hydrogel microchannels lined with a confluent endothelial monolayer. This approach to fabricating microphysiological systems on hydrogel platforms will provide new avenues for technological innovation in disease models, organ-on-a-chip, and personalized medicine.

## Graphical abstract

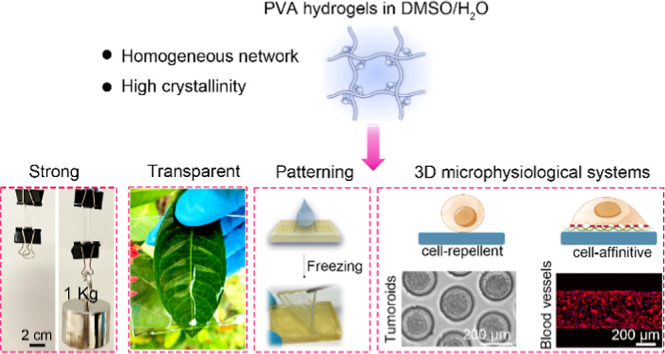

## Introduction

3D microphysiological systems (MPSs) on miniaturized biochips have emerged as powerful platforms for biological research, drug development, and clinical applications by replicating key physiological features of native tissues.^1–3^ Through the integration of tissue engineering, biomedical science, and microfabrication technologies, MPSs enable the assembly of cells and biomaterials into 3D constructs that preserve tissue-specific characteristics, support intercellular interactions, and mimic biomechanical and biochemical cues.^4–7^ MPSs provide a more physiologically relevant microenvironment compared to conventional 2D cultures while offering a faster, cost-effective, and scalable alternative to animal models,^8^ helping to reduce reliance on animal testing and improve clinical translation. By bridging the gap between traditional in vitro systems and in vivo animal models, MPSs on biochips serve as a cutting-edge tool for diverse applications in biomedical research. With rapid advances in MPSs, the design of biochips is undergoing sophistication and multifunctionalization to accommodate increasingly complex biological microenvironments.^9,10^ Polydimethylsiloxane (PDMS), a widely used material in biochip fabrication, remains a preferred choice due to its durability and compatibility with microfabrication techniques.^11^ Recent innovations in PDMS microstructure design have endowed biochips with the ability to recreate complex in vivo-like microenvironments, including oxygen gradients^12^ and functional structures such as vascularized human liver,^13^ blood–brain barrier systems,^14^ and tumor-associated lymphatics,^15^ enabling the successful generation of various MPS models.

As an alternative to the conventional anhydrous elastomer of PDMS, water-rich hydrogel materials are emerging as novel body materials for biodevices.^16,17^ Benefiting from the extracellular matrix-like attributes of high water contents and intrinsically porous structures,^18–20^ hydrogels show great biocompatibility and a variety of tunable properties regarding surface physicochemistry and mechanical characteristics, providing favorable in vitro environments customized for 3D cell culture.^21,22^ In addition, the permeability of hydrogels allows cells in 3D culture with dynamic nutrient and gas exchange,^23^ contributing to more physiologically relevant and functional MPSs. For hydrogels to be processed into free-standing 3D microstructures for functional devices, it is essential for them to possess sufficient mechanical modulus, elasticity, and toughness.^24^ This is because weak hydrogels may cause the collapse of as-prepared structures during critical processes such as demolding and cell culture, particularly in the absence of rigid supporting substrates (e.g., PDMS or acrylic), which compromises both the architectural fidelity of large-scale 3D microstructures and the reproducibility of flow patterns within hollow microchannels. However, traditional designs of strong hydrogels typically involve complex polymeric networks^25^ or nanomaterial incorporation,^26,27^ which may weaken the processability and transparency of hydrogels and thus impede their widespread use in device fabrication for the scientific community. Therefore, developing simple approaches that can produce high-performance hydrogels through straightforward processing techniques—while also meeting the demands of microfabrication for 3D constructs—holds great promise for advancing the use of hydrogel materials in biodevice applications.

Here, we report strong and transparent hydrogel elastomers (STHEs) from poly­(vinyl alcohol) (PVA) networks with favorable manufacturability for biodevices. PVA hydrogels gelled by freeze–thaw cycles undergo solvent-mediated crystallization with distinct network microstructures and properties using water solutions with and without dimethyl sulfoxide (DMSO) (Figure 1a). PVA dissolved in a mixed solvent of DMSO and water can be extensively cross-linked by crystalline domains through one freeze–thaw cycle, leading to the resulting STHEs with superior mechanical properties (Figure 1b) and transparency (Figure 1c), which are both critical for their performance in biodevices. In contrast, those hydrogels prepared from PVA solutions in pure water show much weaker mechanical properties and optical opacity, highlighting the important role of solvent components for the resulting hydrogels. As the gelation of PVA hydrogels is subjected to a simple temperature-controlled process, patterning of STHEs can be easily achieved by replicating structures from molds prepared by commercial techniques of photolithography or 3D printing (Figure 1d; Figure S1, Supporting Information). This process is compared favorably to the traditional microfabrication of PDMS in the soft lithography technique. Here, 2D pattern molds were fabricated on SU-8 photoresist by standard photolithography, while 3D pattern molds were created on photo-cross-linked resin by 3D printing. Additionally, the intrinsic permeability of hydrogels allows STHEs to be readily modified through molecular engineering strategies for customized physicochemical properties, such as controllable deswelling/swelling properties and tailored surfaces of tunable cell affinity (Figure 1e). Our facile approach for 3D microarchitected hydrogels with outstanding mechanical and optical properties provides a versatile platform to fabricate microphysiological systems for a broader scientific community.

**Figure 1:**
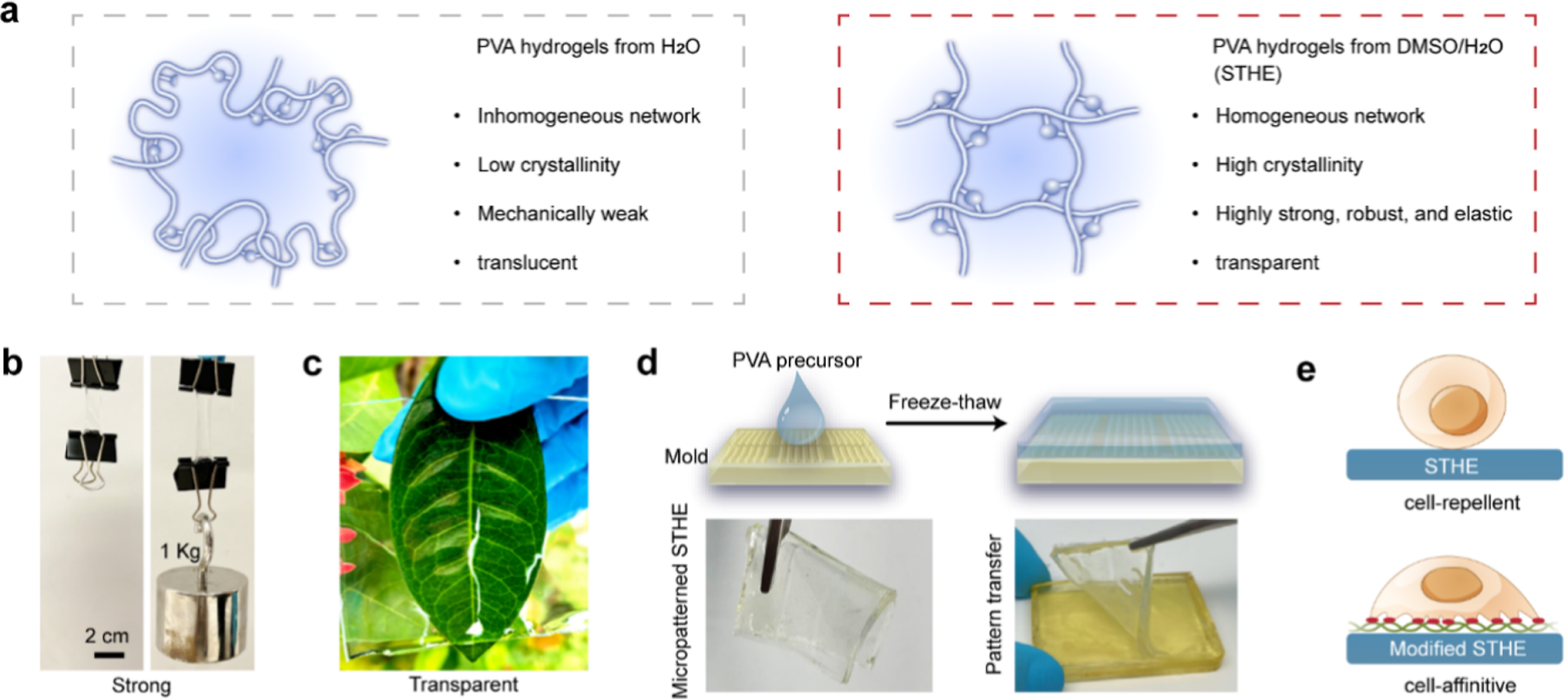
Strong and transparent hydrogel elastomers (STHEs). (a) Schematics of PVA hydrogels derived from precursors in pure water and in the mixed solvent. (b) Photographs of a STHE strip (2 mm thickness) withstanding a weight loading of 1 kg. (c) A photograph of a STHE sample showing high transparency. (d) Microfabrication of STHEs. (e) Schematics of a cell-repellent surface for pristine STHEs and a cell-affinitive surface for STHEs after modification.

## Results and Discussion

### Fabrication and Properties

PVA hydrogels can be readily prepared from PVA solutions in pure water through several freeze–thaw cycles.^28–30^ During exposure of PVA solutions to a low temperature, phase separation proceeds as water freezes, leading to the formation of PVA-rich regions where PVA polymers gradually rearrange into crystallites through intermolecular hydrogen bonds.^31,32^ These crystallites remain intact during the subsequent thaw process, serving as stable physical cross-links for the resulting PVA hydrogels. Noteworthily, PVA with high molecular weight (Mw: 146,000–186,000) and a high degree of hydrolysis (99%+ hydrolyzed) was adopted in this work, as rich hydroxy groups dangling on PVA long chains are beneficial for their intermolecular physical interactions.^27^ According to our initial study (Figure 2a; Figure S2a, Supporting Information), PVA hydrogels derived from the PVA solution (15 wt %) in pure water after one freeze–thaw cycle show a tensile modulus and strength at ∼5 kPa and ∼0.02 MPa, respectively. These values increase with the cycle number of freeze–thaw treatment, reaching ∼42 kPa and ∼0.2 MPa after four cycles, respectively. Further increase of the cycle number has minimal effects on the tensile behaviors of the resulting PVA hydrogels (Figure S3, Supporting Information), indicating maximum levels of crystallization achieved according to this method.^33^

**Figure 2:**
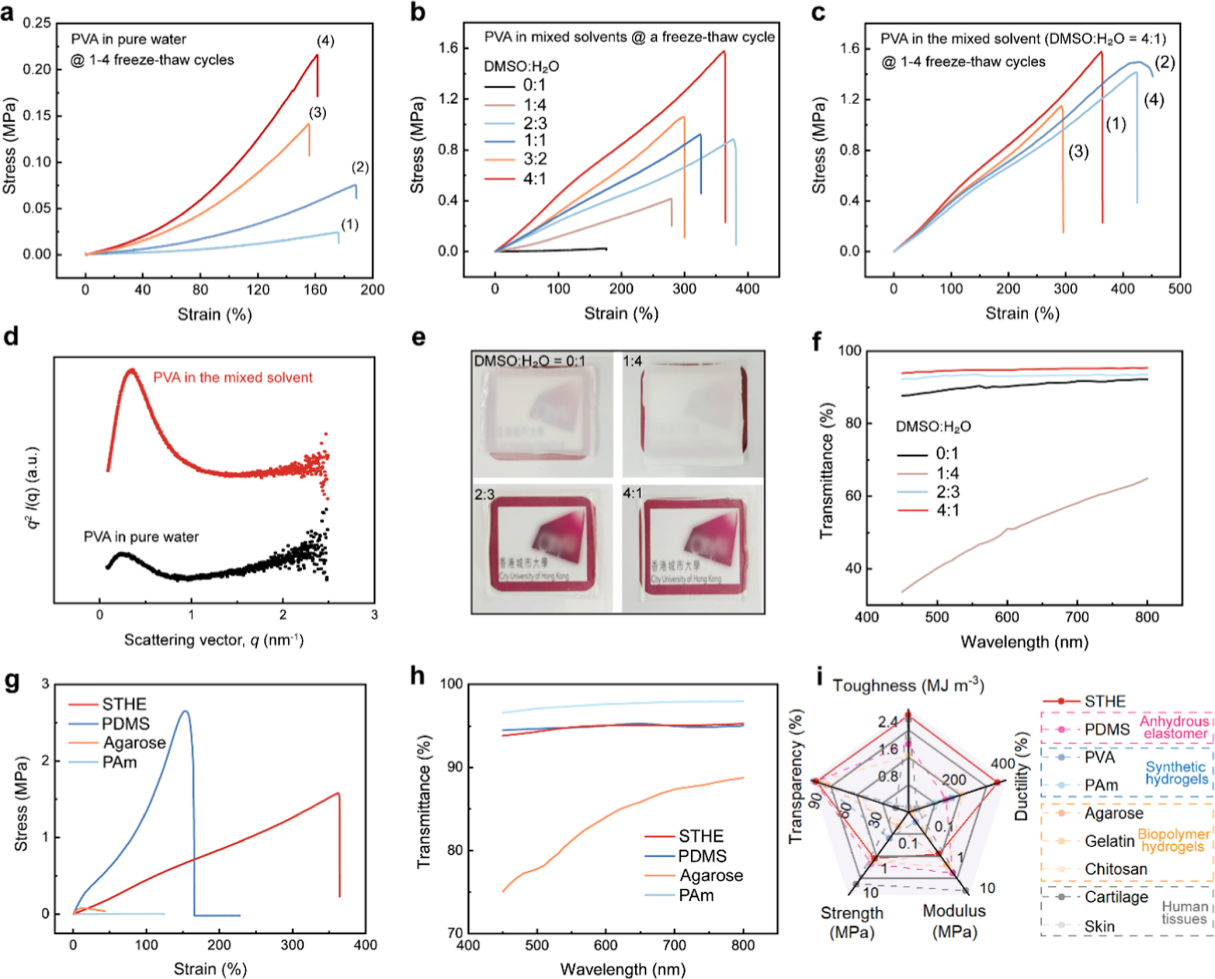
Fabrication and properties. (a) Tensile responses of PVA hydrogels fabricated from PVA dissolved in pure water with different cycle numbers of freeze–thaw treatment. (b) Tensile responses of PVA hydrogels fabricated from PVA dissolved in mixed solvents with one freeze–thaw cycle. (c) Tensile responses of STHEs with different cycle numbers of freeze–thaw treatment. (d) Small-angle X-ray scattering tests. (e) Photographs showing the variation in the transparency for PVA hydrogels (3 mm thickness). (f) Transmittance of PVA hydrogels. (g) Tensile responses of STHEs and other typical polymeric materials. (h) Transmittance of STHEs and other typical polymeric materials. (i) Comparison of STHEs with other common polymeric materials. All experiments were performed on PVA hydrogels after complete solvent exchange, where DMSO was thoroughly replaced with water.

In contrast, PVA hydrogels prepared from PVA solutions in mixed solvents of DMSO and H_2_O show distinct tensile behaviors (Figure 2b; Figure S2b, Supporting Information). In this scheme, the solid content of PVA solutions was also kept constant at 15%, while the mass ratios between DMSO and H_2_O in the solvents were tuned from 0:1 gradually to 4:1. After one cycle of freeze–thaw treatment, the resulting PVA gels in diverse solvents were soaked in DI water to remove residual DMSO, leading to a series of PVA hydrogels for further comparison. The solvent exchange process caused a slight shrinkage of the resulting PVA hydrogels, with the final water content of the hydrogels at ∼80%. Interestingly, a small amount of DMSO in the mixed solvent (20 wt %) led to a significant improvement in the mechanical properties of the resulting PVA hydrogels, with a tensile modulus and strength at ∼92 kPa and ∼0.4 MPa, respectively. At an optimum weight ratio between DMSO and H_2_O of 4:1, the resulting PVA hydrogels showed a high tensile modulus and strength of ∼0.4 MPa and ∼1.6 MPa, respectively. Furthermore, PVA hydrogels were also able to withstand compressive strain over 90%, with a compressive modulus of ∼31 kPa for those from pure water and ∼0.83 MPa for those from the optimum mixed solvent (Figure S4, Supporting Information). A further increase in the contents of DMSO in the mixed solvents rendered a decrease in the mechanical performance of hydrogels, with the final PVA hydrogels derived from PVA solutions in pure DMSO showing a semigel state and a quite low modulus. The excessive amount of the good solvent DMSO will inhibit the cross-link formation of crystallites.^34^ Additionally, it is noteworthy here that only one cycle of freeze–thaw treatment is sufficient to generate the high-performance PVA hydrogels using this optimum mixed solvent (Figure 2c), in contrast with PVA hydrogels prepared from pure water, where time-consuming multiple freeze–thaw cycles were requisite to guarantee their mechanical performance.

The elastic stiffness of polymeric hydrogels is expected to have a positive relation with the cross-linking density.^35^ For this hydrogel system consisting of a single polymeric network with crystallites as cross-links, PVA hydrogels from the mixed solvent have a high tensile modulus (∼0.4 MPa), nearly 10-fold the value for those from pure water at the same solid content (∼42 kPa), providing initial evidence that physical cross-linking is intensified by the mixed solvent during gelation. More definite proof was afforded by small-angle X-ray scattering (SAXS) tests, where scattering intensity (*I*) was recorded as a function of scattering vector (*q*) (Figure S5, Supporting Information). After Lorentz correction, the plots peaked at 0.25 nm^–1^ and 0.34 nm^–1^ (Figure 2d), corresponding to the average intercrystal distances at 25.1 and 18.5 nm for PVA hydrogels from pure water and the mixed solvent, respectively.^36,37^ This discrepancy can be explained by solvent effects on PVA polymers before and after physical cross-linking. As H_2_O is a poor solvent for PVA, PVA tends to fold into coiled chains in pure water due to the rich intrapolymer hydrogen bonding.^34^ However, DMSO, a good solvent for PVA, facilitates a stretched conformation of PVA polymers, enhancing their interpolymer interactions to form a homogeneous 3D network.^38^ Furthermore, the addition of DMSO into the mixed solvents could inhibit the ice crystal growth to large sizes during freezing^39^ and, thus, decrease the average distance between PVA-rich regions, which also contributes to an increase in the crystallite density for PVA hydrogels from the mixed solvent.

Improved network topology and intensified physical cross-linking not only impart a marked effect on the mechanical performance but also influence the transparency significantly (Figure 2e,f; Figure S6, Supporting Information). PVA hydrogels from pure water with the presence of inhomogeneous aggregation domains^33^ were translucent with visible-light (450–800 nm) transmittance through a film sample (1 mm thickness) at ∼90.5%. A low fraction of DMSO in the solvent (20 wt %) led to PVA hydrogels turning into an almost opaque state, and the transmittance of hydrogels grew with further increases in the DMSO contents in the mixed solvent, with highly transparent PVA hydrogels (94.8% transmittance) at the optimal DMSO-to-H_2_O weight ratio of 4:1. The optical anomaly at a DMSO concentration of 20 wt % may be attributed to the reduced solvation of the mixed solvent at a specific DMSO-to-H_2_O ratio.^40^ Here, we refer to the highly transparent, strong, and elastic hydrogels (Figure S7, Supporting Information) as STHEs. Notably, the STHEs demonstrate remarkable stability, maintaining both mechanical properties and optical transparency with negligible changes over a 7 day period (Figure S8, Supporting Information). Finally, a comprehensive comparison of mechanical performance and transparency was performed among STHEs, the anhydrous elastomer of PDMS, and other typical synthetic and natural hydrogels (Figure 2g,h). To consider the contribution of both elastic stiffness and ductility to mechanical performance, energy dissipation during tension until fracture was recorded to estimate material toughness, as determined by the area under tensile curves.^41,42^ STHEs exhibited a toughness of ∼2.82 MJ m^–3^ and PDMS at 1.99 MJ m^–3^, 2 orders of magnitude higher than typical hydrogels of agarose and polyacrylamide (PAm). STHEs also showed similarly high transparency to PDMS, slightly lower than that of PAm but far outperforming that of agarose. Indeed, the performance of STHEs is distinguishable from that of common polymeric materials (Figure 2i; Table S1, Supporting Information). As soft elastomers, STHEs have mechanical moduli consistent with the natural soft tissue of human skin, but their toughness can even compare to that of the natural hard tissue of cartilage.

### Mechanical Comparison

The resistance of materials toward cracking and deformation, which can be identified by fracture energy and shape recovery, respectively, is critical for the robustness and durability of fabricated devices. We compare the mechanical performance of water-rich STHEs with that of anhydrous PDMS, which is widely adopted in microfabrication for lab-based biodevices. The fracture energy of PDMS is about 465–522 J m^–2^, as determined by both tear tests and pure shear tests (Figure 3a–c). In comparison, STHEs, although with a water content of ∼80%, have a markedly higher fracture energy at 730–864 J m^–2^, as a result of the extensive physical cross-linking and intermolecular interactions, which can provide sufficient resistance upon crack opening. Furthermore, the elasticity, indicated by shape recovery for elastomers, was assessed by multiple tensile cycles. With cyclic loading between 0 and 0.5 MPa, PDMS shows low hysteresis of ∼6%, indicating little energy dissipation during normal load and unload operations^43^ (Figure 3d). Excepting its low hysteresis, the high elasticity of PDMS can also be proved by its almost 100% shape recovery throughout 30 tensile cycles (Figure 3f). Interestingly, STHEs exhibit even lower hysteresis (∼2.9%), and the shape recovery keeps at nearly 100% even at an extension to ∼60% (Figure 3e,f), which is supposed to be attributed to the strong cross-links of dense crystallites. As a result, STHEs have superior toughness and elasticity exceeding PDMS, suggesting their qualification for wide applications in device fabrication from mechanical perspectives.

**Figure 3:**
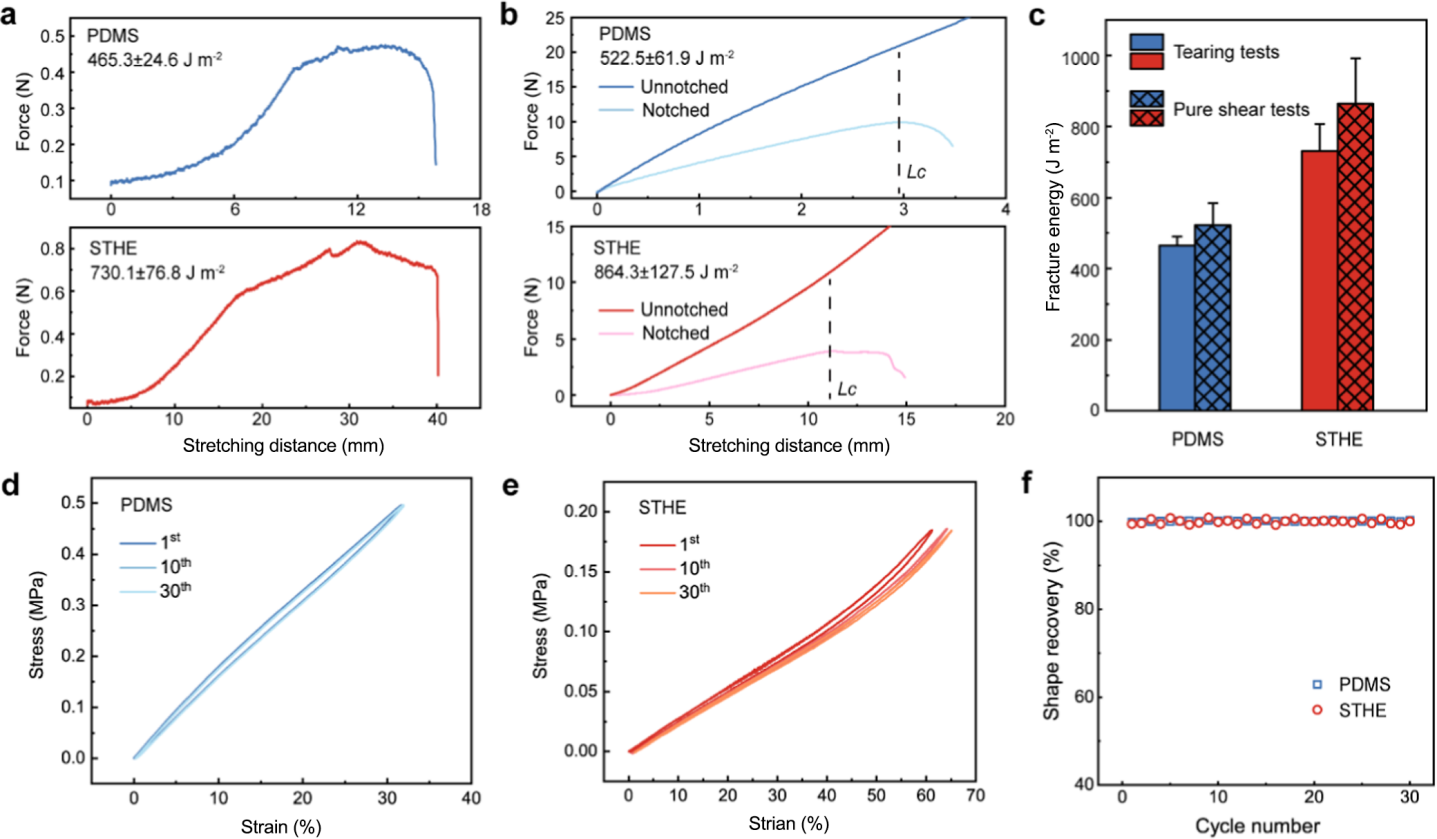
Mechanical comparison between elastomers of PDMS and STHEs. (a,b) Fracture energy tested by both tear tests (a) and pure shear tests (b). (c) Comparison of the fracture energy between two elastomers. (d,e) 30 cycles of tensile loading and unloading for PDMS (d) and STHEs (e). (f) Comparison of shape recovery between two elastomers.

### Microfabrication

The simplicity of the processing steps allows for the microfabrication of STHEs into diverse configurations by facile pattern transfer. First, precise planar micropatterns of photoresist on silicon wafers were achieved by standard photolithography technologies.^26^ STHE precursors were then poured atop wafer molds to replicate these patterns through the solidification process of a freeze–thaw cycle (Figure 4a). The resulting gels could be easily peeled off from stiff substrates with high fidelity due to their outstanding mechanical properties against cracking and permanent deformation, as aforementioned. This property distinguishes them from PVA hydrogels cross-linked via solvent exchange, which typically exhibit strong adhesion to substrates.^44^ A variety of patterns, including dots, lines, serpentine curves, stars, and snowflakes, were successfully transferred from the molds with an accuracy reaching ∼96% in comparison with the original features of photomasks (Figure 4b). Notably, patterns with complicated features (rich in angles) and tiny dimensions (20 μm resolution) can be precisely transferred according to our method, expanding the materials toolbox of microstructured elastomers, which currently are restricted within quite limited options like PDMS. It is noteworthy that the solvent exchange process to exclude residual DMSO will lead to gel shrinkage with a final volume change of ∼28.5% (Figure 4c). In cases where pattern sizes should be strictly controlled, a small amount of superhydrophilic polymer of PAm (3 wt %) can be incorporated into the PVA network to effectively inhibit the deswelling of hydrogels in water (Figure 4c). The controllable swelling properties of hydrogels can also be applied in smart actuation for programmable surface wrinkles and other application scenarios.^45^ However, the incorporation of polyacrylamide to counteract hydrogel shrinkage elicits a slight decrease in mechanical properties of the resulting hydrogels (Figure S9, Supporting Information).

**Figure 4:**
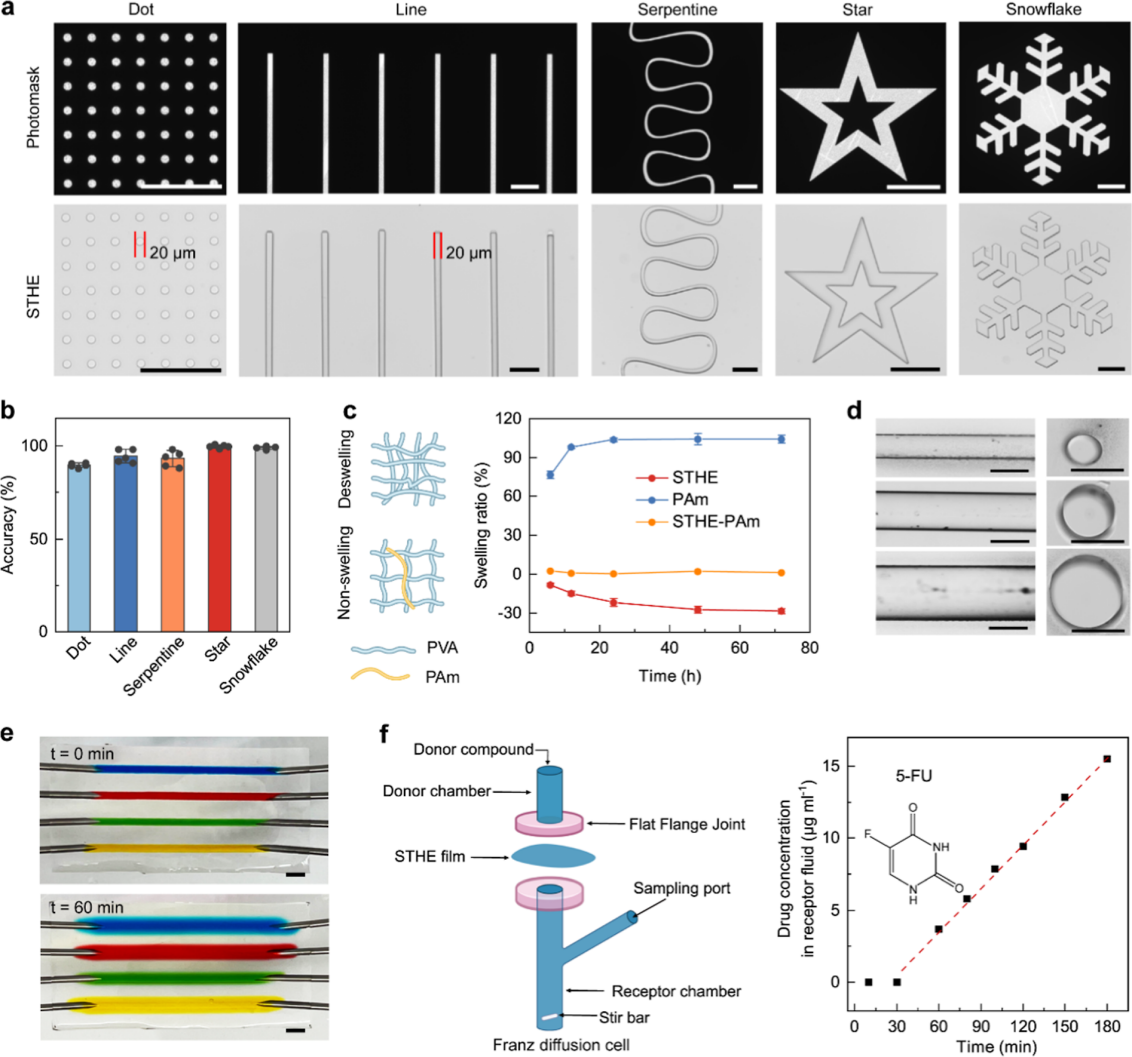
STHE microfabrication. (a) Brightfield images of photomask and microfabricated STHE patterns accordingly. Scale bar: 200 μm. (b) Accuracy of pattern features for microfabricated STHEs. (c) Controllable swelling properties of STHEs by composite with hydrophilic polymer of polyacrylamide (PAm). (d) STHEs with hollow microchannels of diameters of 300, 500, and 700 μm, respectively. Scale bar: 500 μm. (e) A STHE with 4 parallel channels (1 mm diameter) infused with water-soluble dyes for 60 min. Scale bar: 2 mm. (f) Permeability of STHE for an antitumor drug of 5-fluorouracil (5-FU).

Stereo patterns of STHEs, as exemplified by linear hollow microchannels, were also constructed, benefiting from the superior mechanical properties to support free-standing 3D constructs. Needles with diverse diameters were preplaced into STHE precursors. After the solidification and the solvent exchange, needles were removed to generate channels with diameters of 300, 500, and 700 μm, respectively (Figure 4d). A marked attribute of STHEs, distinct from anhydrous PDMS, is their permeability for small molecules, providing potentials in drug delivery and onsite biodetection of biochemical molecules within biochips.^46,47^ As qualitative proof, after infusion of water-soluble dyes into STHE channels for 60 min, dyes penetrated the hydrogel walls and permeated gradually into the hydrogel matrix (Figure 4e). For further understanding the diffusion of small molecules in STHEs, a typical antitumor drug 5-fluorouracil (5-FU) was used in a custom-made setup of a Franz diffusion cell (Figure 4f). The drug diffused from an upper donor chamber to a bottom receptor chamber through an STHE film positioned in between. According to the time-dependent increase of the drug concentration in the receptor chamber (Figure 4f; Figure S10, Supporting Information), the permeability coefficient of STHEs for 5-FU can be determined at 1.8 × 10^–3^ cm h^–1^, orders of magnitude higher than those for natural skin tissues,^48^ suggesting the ability of efficient mass transfer within bulk STHEs. The good permeability of hydrogels not only supports cell survival but also allows for the establishment of specific gradient profiles based on diffusion, which can be used to study directed cell migration in response to chemotaxis assays.^49^

### 3D Microphysiological Systems

Interactions between STHEs and cells were initially investigated by studying the biocompatibility and cellular affinity of the STHEs. STHEs showed great cytocompatibility by the cytotoxicity tests (Figure S11, Supporting Information), indicating their suitability for biodevice manufacturing. Furthermore, the surface of pristine STHEs is quite cell-repellent, in contrast with PDMS where a significant number of cells attached on its surface (Figure 5a). This behavior may arise from the overhydrophilic surface of STHEs with a lack of cell-adhesive ligands.^50^ Similar attributes were observed in other hydrogel materials such as agarose and PAm. The intrinsic anticell-adhesive properties of STHEs are favorable to be adopted to produce tumor spheroids for in vitro tumor models, where tumor cells self-organize into 3D aggregates resembling their in vivo state.^51^ Beyond the surface biochemistry, topography of STHEs is another critical factor interfering with spheroid morphogenesis.^52^ Depending on the aforementioned facile pattern-transfer approach, STHE microwells (230 μm diameter) with precise 3D constructs of both flat and U-shaped bottoms were microfabricated according to 3D-printed resin molds (Figure 5b,c). After lung tumor cell seeding, cells initially aggregated due to gravitational contributions in U-shaped microwells, facilitating the formation of well-packed spheroids after 3 days of culture (Figure S12, Supporting Information). In contrast, tumor cells distributed randomly after seeding in flat-bottom microwells, which is adverse for spheroid morphogenesis with several loose aggregates in each microwell after culture. Additionally, initial cell seeding density also markedly influenced the final morphology of tumor spheroids. Experimental results confirmed that an optimal seeding density of 50 cells per microwell produced round and uniformly sized spheroids (Figure S13, Supporting Information).

**Figure 5:**
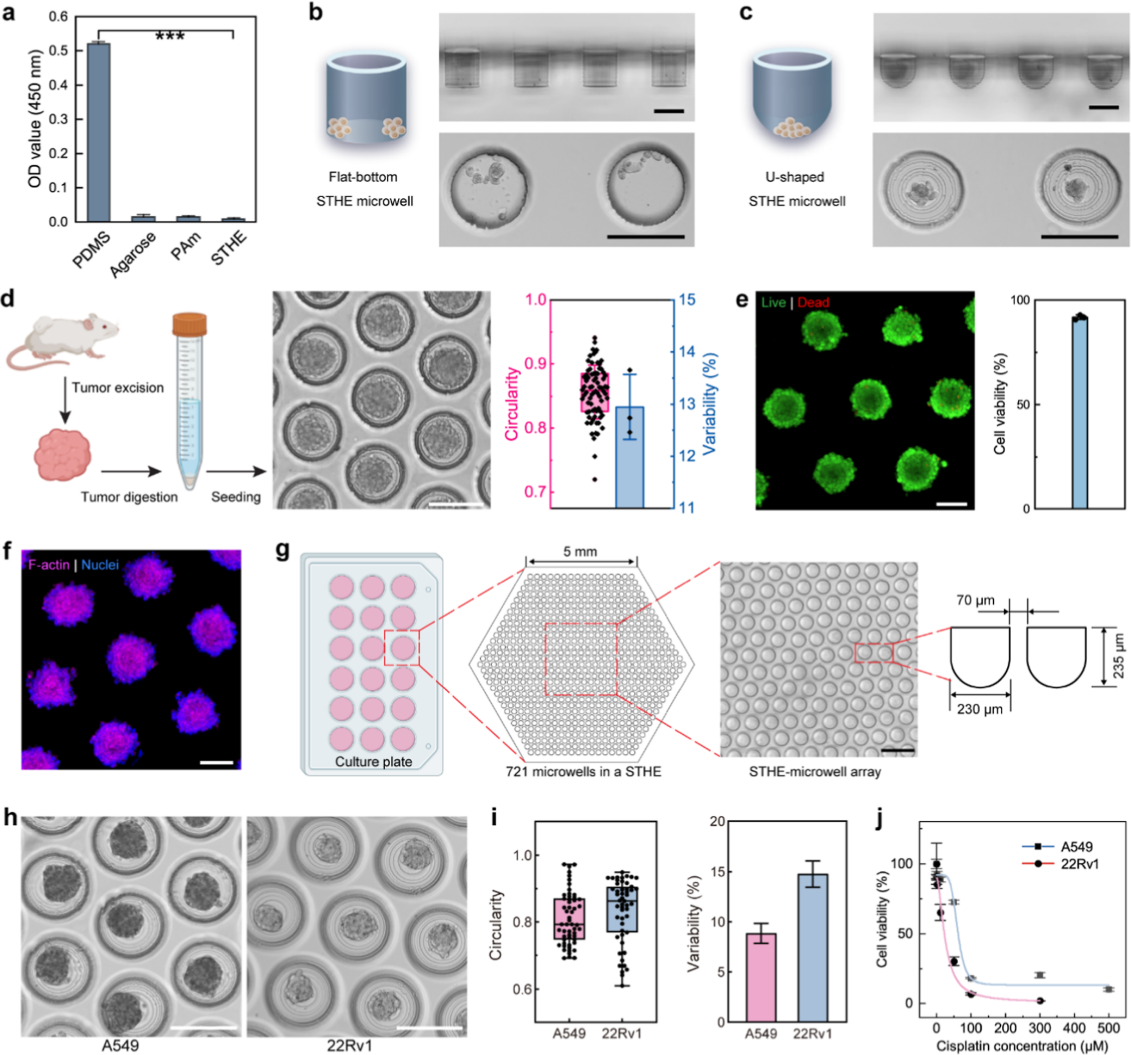
In vitro tumor models. (a) Cell attachment on different types of substrates (***p < 0.001 by Student’s *t*-test). (b,c) STHE microwell arrays with flat (b) and U-shaped (c) bottoms for tumor-spheroid generation. (d) Workflow of producing mouse tumor tissue-derived spheroids in microwell arrays. Created in BioRender. Brightfield images show spheroids with high circularity and low variability after 3 days of culture. Scale bar: 200 μm. (e) Cell viability of the spheroids determined by Live/Dead staining (live cells in green and dead cells in red). Scale bar: 100 μm. (f) Immunofluorescent staining of F-actin (in purple) and nuclei (in blue) for the spheroids. Scale bar: 100 μm. (g) Schematic illustration of the design of microwell arrays of STHEs. Created in BioRender. A brightfield image shows the configuration of microwells in STHEs. Scale bar: 500 μm. (h) Brightfield images of spheroids of A549 and 22Rv1 in the microwell arrays after 3 days of culture. Scale bar: 200 μm. (i) Circularity and size variability of the spheroids after 3 days of culture. (j) Dose response of the spheroids regarding the anticancer drug cisplatin.

An in vitro tumor model of spheroids was generated by culturing primary cells derived from lung cancer mice in U-shaped STHE microwells (Figure 5d). After 3 days of culture, the resulting spheroids of ∼138 μm in diameter (Figure S14, Supporting Information) had a high circularity at ∼0.86 with a low variability at ∼12.9%. The high transparency of STHEs allowed in situ observation of spheroid viabilities by Live/Dead staining, showing a high survival rate of ∼91.8% for cells within spheroids (Figure 5e; Figure S15, Supporting Information). Furthermore, cancer cells in the spheroids also showed fast maturation, as evidenced by the pronounced expression of the cytoskeletal protein F-actin^53^ (Figure 5f), making them well-suited for in vitro cancer research applications. As a demonstration of practical applications, spheroids from two cancer cell lines, A549 and 22Rv1, were produced accordingly in STHE microwell arrays (721 microwells in a hexagonal STHE with a 5 mm side length) for high-throughput drug screening (Figure 5g). The resulting A549 and 22Rv1 spheroids in microwells showed uniformly round shapes, with overall high circularity values at ∼0.81 and 0.83, respectively, and low variability values at 8.9% and 14.8%, respectively, after 3 days of culture (Figure 5h,i; Figure S16, Supporting Information). Both A549 and 22Rv1 spheroids maintained high viabilities above 94% before anticancer drug treatment (Figure S17, Supporting Information), which decreased effectively after exposure to the drug cisplatin (Figure 5j). This test showed A549 and 22Rv1 cells were sensitive to cisplatin, demonstrating their corresponding half inhibitory concentration (IC_50_) values of 61.0 and 24.9 μM, respectively.

A tissue model of blood vessels was also built based on perfusable microchannels in STHEs. To promote cell attachment on the STHE surface, a biopolymer of gelatin was coated on the STHE surface by the penetration of gelatin into the STHE matrix with a shallow layer of ∼180 μm (Figure 6a), according to a simple surface modification method by interpolymer entanglement and chemical bonding.^50,54^ The improved cell adhesion on modified STHEs was observed after seeding primary human umbilical vein endothelial cells (HUVECs) in comparison to pristine STHEs (Figure 6b). Furthermore, as evidenced by F-actin staining for cytoskeletal imaging, the morphology of HUVECs attached on pristine STHEs showed aggregated round spheres similar to the aforementioned case for tumor cells, while HUVECs on modified STHEs spread well into a monolayer (Figure 6c,d). The HUVECs grown on modified STHEs also show great cell–cell junctions indicated by the marked expression of vascular endothelial cadherin (VE-Cadherin), unlike those on pristine STHEs. The favorable surface of modified STHEs for endothelial monolayer formation highlights their potential for constructing in vitro blood vessels (Figure 6e). Vessel models comprising a tubular endothelial monolayer attached to the inner surface of perfusable microchannels in STHEs were successfully built, as evidenced by the 3D reconstructive and cross-sectional fluorescence images of HUVECs, where cells spread uniformly and lined along the inner wall of STHE microchannels (Figure 6f).

**Figure 6:**
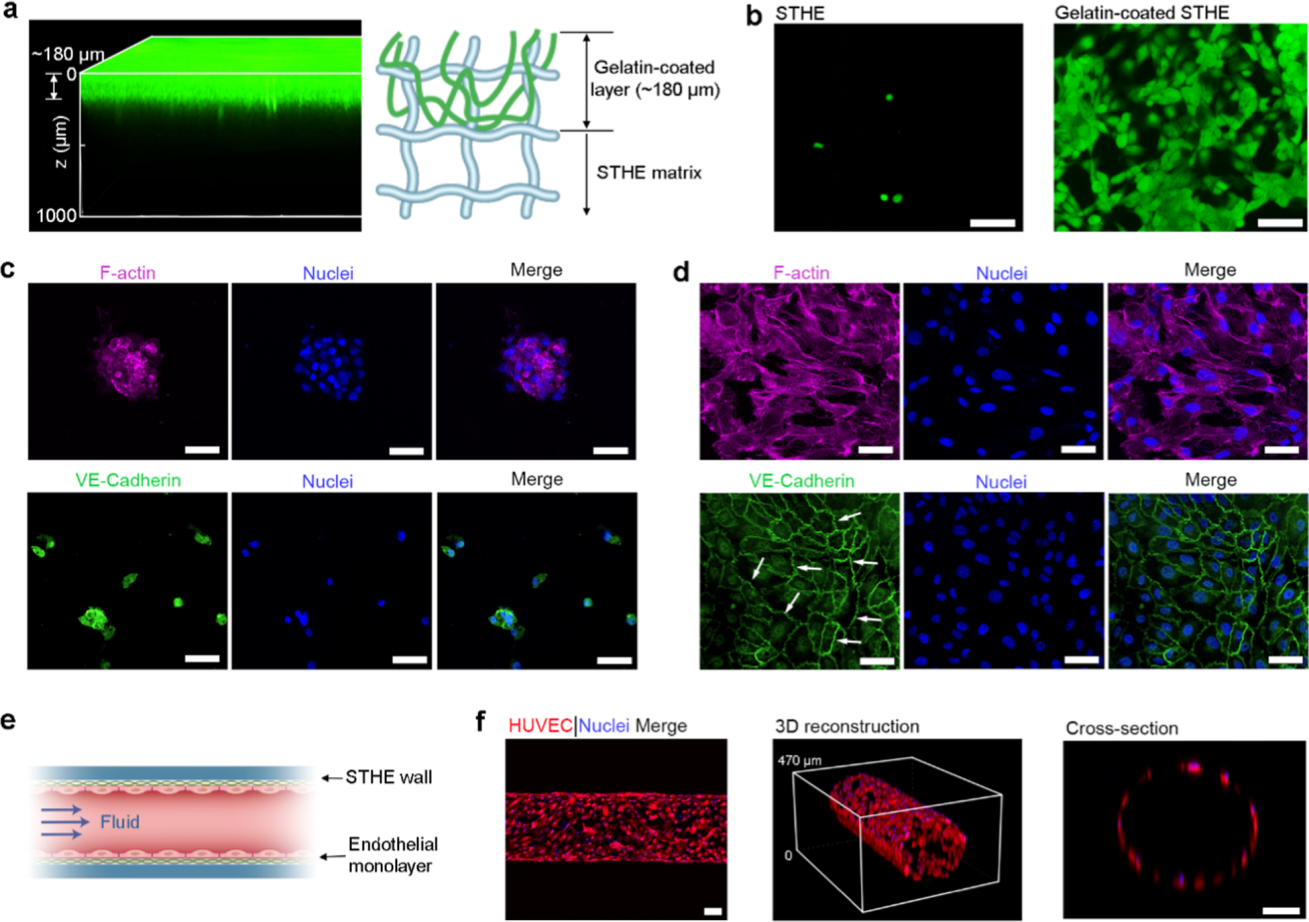
In vitro blood vessel models. (a) STHEs coated with gelatin labeled with green fluorescence. (b) Fluorescence images of HUVECs cultured on STHEs and gelatin-coated STHEs as visualized by Live/Dead staining (live cells in green and dead cells in red). Scale bar: 200 μm. (c,d) Immunofluorescent staining of endothelial-associated proteins (F-actin in purple; VE-Cadherin in green; nuclei in blue) for HUVECs attached on STHEs (c) and gelatin-coated STHEs (d). Scale bar: 50 μm. (e) A schematic of an in vitro blood vessel model based on an STHE microchannel lined with an endothelial monolayer. (f) 3D projections of confocal fluorescence images showing the endothelial monolayer in an in vitro blood vessel model with red-labeled HUVECs. Scale bar: 100 μm.

## Conclusion

In this work, we have demonstrated a rational strategy to facilely fabricate STHEs with a good combination of mechanical robustness, elasticity, and transparency, which can be further readily engineered into elaborate stereo constructs for applications in 3D microphysiological systems. On the other hand, the surface of STHEs can be tailored to be either cell-repellent or cell-affinitive, which are important to satisfy applications in diverse scenarios, as exemplified by the generation of tumor spheroids and endothelial monolayers. The versatility of STHEs holds great promise for their wide use in hydrogel-based biodevices, providing opportunities to research cell/tissue responses in an in vivo-like environment. Further molecular engineering to achieve strong bonding between hydrogels and diverse surfaces^32,55^ will permit the development of a new class of advanced biointerfaces and hydrogel-based microfluidics.^16^ Considering the intrinsic permeability of hydrogels, further integration of minimized smart sensors within STHEs will broaden their functionalities in in situ monitoring of physiological activity through the diffusion of small chemicals rather than relying solely on optical methods. STHEs with optimal multifunctional properties and manufacturability may provide a novel, powerful platform for the design of various hydrogel-based biodevices for precision diagnostic and therapeutic technologies.

## Methods

### Hydrogel Preparation

PVA hydrogels were prepared by freeze–thaw cycles. Specifically, PVA (Mw: 146,000–186,000, 99%+ hydrolyzed; Sigma-Aldrich) was dissolved either in deionized (DI) water at 95 °C overnight by magnetic stirring or in a mixed solvent of dimethyl sulfoxide (DMSO) and DI water with mass ratios between two components at 1:4, 2:3, 1:1, 3:2, and 4:1, respectively, at 120 °C under magnetic stirring for 2 h. The as-prepared PVA solutions (15 wt %) were centrifuged (Centrifuge 5810R, Eppendorf) at 4000 rpm for 1 min to remove bubbles before pouring solutions into molds. Physical cross-linking of PVA was achieved by freezing solutions at −20 °C for 8 h and thawing at 25 °C for 3 h. The residual DMSO was removed from the samples by solvent exchange in DI water for 24 h to generate PVA hydrogels. STHEs were fabricated from the mixed solvent with a DMSO-to-H_2_O weight ratio of 4:1. For comparison, polyacrylamide (PAm) hydrogels were synthesized by photopolymerization of acrylamide (20 wt %; Sigma-Aldrich) using the cross-linker *N*,*N*′-methylenebis­(acrylamide) (0.3 wt %; Sigma-Aldrich) and the photoinitiator IRGACURE 2959 (1 wt %; Sigma-Aldrich) in deaired DI water under UV-light exposure (25 mW cm^–2^; CL-1000 Ultraviolet Cross-Linkers, UVP) for 30 min. STHE-PAm hydrogels were prepared by performing a freeze–thaw cycle upon a PVA (15 wt %) - PAm (3 wt %; Mw: 150,000; Sigma-Aldrich) solution in DMSO and H_2_O (weight ratio of 4:1) mixed solvent, according to one cycle of freeze–thaw treatment. Agarose hydrogels were prepared by cooling agarose solution (7.5 wt % agarose dissolved in boiling water) at 25 °C for 3 h. The anhydrous elastomer of polydimethylsiloxane (PDMS) was fabricated by mixing silicone solution and curing agent (weight ratio of 10:1, Dow Corning) before solidification at 80 °C for 2 h after vacuum degassing.

### Mechanical Tests

Cylinder samples (10 mm diameter and 3 mm thickness) were used for compression tests, with a deformation rate of 50% min^–1^ under a mechanical tester (Zwick Roell). Dumbbell-shaped samples (15 mm in length, 3 mm in width, and 1.5 mm in thickness) were used for tensile tests, with a deformation rate of 100% min^–1^. The modulus was calculated from the initial slope of the stress–strain curves. The fracture energy was calculated by integration of the stress–strain tensile curves. Cyclic tensile loading for 30 cycles was performed between 0 and 0.5 MPa regarding PDMS and between 0 and 0.2 MPa regarding STHEs. The shape recovery (*R*) was calculated as

1 $$R=\left(\varepsilon_{x}-\varepsilon_{y}\right)/\left(\varepsilon_{x}-\varepsilon_{z}\right)\times 100\%$$

whereε_*x*_, ε_*y*_, and ε_*z*_ are the strain after loading, the strain after unloading, and the strain before loading, respectively, for each cycle.

The fracture energy (Γ) was determined by both tearing tests and pure shear tests. For the tearing tests, film samples (8 mm width, 50 mm length, and 2 mm thickness) were cut into a trouser shape with a 20 mm notch. The two arms were secured in a mechanical tester and stretched at a constant rate of 2 mm s^–1^. The Γ was calculated according to

2 Γ=2F/t

where *F* is the steady-state tearing force and *t* is the thickness of the samples. For the pure shear tests, rectangular samples (30 mm width, 20 mm length, and 2 mm thickness) were prepared with a 15 mm notch introduced along the width. The samples were clamped with a fixed separation distance of 15 mm, and force–extension curves were recorded at an extension rate of 2 mm s^–1^. The fracture energy was calculated as

3 $$\Gamma =U\left(L_{C}\right)/\left(w\times t\right)$$

where *w* and *t* are the width and thickness of samples, respectively, and *U*(*L*_C_)­represents the work done by the tensile force on unnotched samples until a critical extension length *L*_C_, defined as the extension distance at which crack propagation begins in the corresponding notched samples.

### Optical Characterization

Small-angle X-ray scattering (SAXS) experiments were conducted on PVA hydrogels fabricated from pure water and the mixed solvent (weight ratio between DMSO and H_2_O at 0:1 or 4:1, respectively). Hydrogel samples with 1 mm thickness were mounted on a SAXS instrument (Xeuss 3.0, Xenocs) with the distance between the sample and detector at 1 m and tested using X-ray with a 1.54 Å wavelength at room temperature. The SAXS data was processed by XSACT software provided by the Xenocs company.

The transparency of samples was determined by the transmittance calculated by the intensity ratio of transmitted to incident visible light. Visible light with wavelengths from 450 to 800 nm was transmitted through film samples with a constant thickness of 1 mm using a UV–visible spectrometer (721, Jinghua Instruments).

### STHE Microfabrication

Microarchitected STHEs were achieved by replicating structures from molds. Precise 2D patterns of SU-8 photoresist (50 μm thickness) on a silicon wafer were fabricated by the standard photolithography approach.^56^ Hot STHE precursors were poured atop the silicon wafer and underwent a freeze–thaw cycle as aforementioned. The resulting free-standing PVA gels were peeled off from the substrate and then soaked in DI water for solvent exchange to generate microarchitected STHEs. Precise 3D structures of a photo-cross-linked resin were fabricated via a high-quality 3D printing machine (nanoArch S140, BMF Precision Tech Inc.) with a layer thickness of 10 μm. 3D-printed micropillar arrays (230 μm diameter, 235 μm height) were used as the molds for fabricating microwell STHEs through the aforementioned pattern-transfer approach. In addition, stainless steel needles with diverse diameters were used as the molds to fabricate STHEs with hollow microchannels.

### Swelling and Permeability Tests

The swelling properties were researched by measuring the volume of gels before and after a phosphate-buffered saline (PBS) bath. After cross-linking, samples (10 × 10 × 1 mm^3^) were soaked in PBS for 3 days at room temperature to reach their swelling equilibrium. The swelling ratio of gels was calculated by dividing the volume change during this process by the initial volumes.

The permeability of STHEs was researched by characterization of the diffusion of the 5-FU drug (HY-90006, MedChemExpress) through hydrogel films using a custom-made Franz diffusion cell (orifice diameter of 9 mm). First, the standard curve of the solution concentrations and UV light absorbance at 266 nm was confirmed by a microplate reader (BioTek Synergy H1) using 5-FU solutions in DI water (200 μL). After that, STHE films (1 mm thickness) were used to separate the upper donor chamber filled with 5-FU solution (5 mg mL^–1^, 0.6 mL) and the bottom receptor chamber filled with DI water (5.6 mL) (Figure 4f). The liquid in the receptor chamber was magnetically stirred, and 50 μL of the liquid was extracted at a fixed interval of 30 min using a syringe before refilling the receptor chamber with an additional 50 μL of DI water to keep the liquid volume constant. The 5-FU concentration of the extracted liquid was determined by a microplate reader. The permeability coefficient (*K*) of STHEs was calculated by

4 $$K=J_{S‐S}/c$$

where *C* is the concentration in the donor chamber and *J*_S‑S_ is the steady-state flux (μg cm^–2^ h^–1^) calculated by dividing the amount of drug diffused through the STHE film by the diffusion time and the exposed area of the STHE film (i.e., 0.64 cm^–2^).

### Cell Culture

Nonsmall lung cancer cell A549 and Lewis lung carcinoma LLC cells (ATCC) were cultured in high-glucose Dulbecco’s modified Eagle’s medium (DMEM, Gibco) supplemented with 10% fetal bovine serum (FBS; Gibco) and 1% penicillin–streptomycin antibiotics (Gibco). Prostate cancer cells 22Rv1 were cultured in RPMI 1640 medium (Gibco) containing 10% FBS and 1% penicillin–streptomycin. Red fluorescent protein (RFP)-expressing human umbilical vein endothelial cells (HUVECs; Lonza) were cultured in Endothelial Growth Medium (EGM-2, Lonza) and used up to passage 7.

Biocompatibility of STHEs was confirmed using the conditioned medium collected from endothelial growth medium incubated with STHEs for 24 h at 37 °C. HUVECs were cultured with the conditioned medium and normal medium (control groups), respectively. The CCK-8 assay kit (HY-K0301, MedChemExpress) was used to calculate the cell proliferation after culture. Furthermore, cell adhesion on diverse samples was also characterized. Cell suspension (1 × 10^4^ A549 cells, 500 μL) was seeded atop disk-shaped samples (10 mm diameter, 1 mm thickness) and incubated overnight. Nonattached cells were gently washed away by PBS, and the rest of the cells were then quantified by a CCK-8 assay.

### Experiments on In Vitro Tumor Models

Primary cancer cells were harvested from tumor tissues of male C57/BL6 mice (5 months of age) 4 weeks after injecting LLC cells into the right flanks of mice. After dissociation of tumor tissues into single cells using the PythoN Tissue Dissociation Kit (Singleron PythoN), the cell suspension was used for further experiments. The animal experiments were performed in accordance with the Animal Care and Ethics Committee of City University of Hong Kong (A-0528).

Microwell STHEs were sterilized in 70% ethanol solution for 1 h before immersion in PBS for 1 day to remove the redundant solvents. The resulting microwell STHEs were placed into tissue culture plates before cell seeding of A549 cells at a low concentration of about 15 cells per microwell to research the variation of spheroid generation in flat-bottomed and U-shaped microwells. Otherwise, the primary cancer cells, A549 and 22Rv1 cells, were seeded into U-shaped STHE microwell arrays at an optimum cell density of 50 cells per microwell to produce batch spheroids after 3 days of incubation. In order to facilitate cell aggregation, each plate was centrifuged for 5 min at 1500 rpm after cell seeding.

Bright-field images were obtained to measure the spheroid circularity and area. At least 50 spheroids were counted by using Nikon NIS-Elements software to determine the circularity and area. The spheroid circularity was calculated by

5 $$Circularity=4\pi A/P^{2}$$

where *A* and *P* are the area and perimeter of tumor spheroids, respectively.^57^ The variability to assess the spheroid uniformity was estimated by the percentage of the standard deviations relative to the means for the spheroid area.

Fluorescence images of cell viability were obtained by staining spheroids in a working solution (1 mL) comprising Calcein-AM (green fluorescence; 1 μL; Beyotime) and propidium iodide (red fluorescence; 1 μL; Beyotime) at 37 °C for 30 min before washing with buffer three times. Z-stack fluorescence images were captured at 7 μm intervals under a scanning laser confocal microscope (Eclipse Ni, Nikon) and merged with Max Projection for analysis. Cell survival in tumor spheroids was determined by

6 $$Viability=A_{L}/\left(A_{L}+A_{d}\right)\times 100\%$$

where *A*_L_ is the area of live cells (green color) and *A*_d_ is the area of dead cells (red color) in the fluorescence images. To visualize the cytoskeleton of mouse tumor spheroids, the tissue-derived spheroids were fixed with 4% paraformaldehyde (28,906, Thermo Fisher) for 10 min at room temperature, followed by washing with PBS. After that, the samples were stained with phalloidin-iFluor 647 (1:1000, ab176759) in 1% bovine serum albumin (BSA; A2153, Sigma-Aldrich) for 1 h. Cell nuclei were stained blue with DAPI (R37606, Invitrogen).

Stock solutions of cisplatin (50 mM; HY-17394, MedChemExpress) in *N*,*N*-dimethylformamide (DMF; 68–12–2, Sigma-Aldrich) were diluted in culture medium to yield varied concentrations of 0.1, 1, 10, 50, 100, 300, and 500 μM for testing the dose responses for A549 and 22Rv1 tumor spheroids. Drug-containing mediums (500 μL) were loaded into 48-well plates and incubated with spheroids for 48 h before cell survival analyses. Blank medium with pure DMF is used for a control. Subsequently, the cell survival was determined using a CellTiter-Luminescent 3D cell viability assay kit (C0061M, Beyotime). The relative IC_50_ values of cisplatin were determined based on dose–response curves using GraphPad Prism 10.

### Experiments on In Vitro Blood Vessel Models

STHEs with cell-affinitive surfaces were achieved by coating with fish gelatin (G7041, Sigma-Aldrich).^50,54^ First, STHEs were soaked in glutaraldehyde solution (2 wt %) for 15 min to introduce aldehyde groups to PVA chains and rinsed thoroughly with PBS to remove excessive glutaraldehyde. After that, the resulting STHEs were soaked in gelatin solutions (20 wt %) for 5 min to form a penetrated gelatin-coated layer. HUVECs (5 × 10^4^ cells per well) were seeded onto sample surfaces to evaluate cell adhesion on STHEs before and after modification.

STHE microchannels with an inner diameter of ∼380 μm were fabricated by embedding 25 G needles in the precursors. After the solidification and the solvent exchange, the surface of microchannels was coated with gelatin before being soaked in EGM-2 medium overnight. RFP-labeled HUVECs (3 × 10^6^ cells per mL) were infused into the microchannels before incubation at 37 °C for 1 h for HUVEC attachment. Subsequently, the device was flipped 180°, and the cell suspension was reintroduced. This process was repeated two times to ensure homogeneous cell coverage on the inner surface of the microchannels. The in vitro blood vessel model was maintained with continuous medium flow (0.l mL h^–1^).

For immunofluorescent staining, the cells, at room temperature, were fixed with 4% paraformaldehyde for 10 min, permeabilized with 0.1% Triton X-100 (X100, Sigma-Aldrich) for 15 min, and blocked with 4% BSA (A2153, Sigma-Aldrich) and 10% goat serum (G9023, Sigma) for 3 h. Then, the fixed cells were incubated with primary antibodies of VE-Cadherin (1:200, ab33168) overnight at 4 °C. After washing thoroughly with PBS three times, cells were incubated with Alexa Fluor 488 AffiniPure goat antirabbit antibody (1:200, Jackson ImmunoResearch Laboratories) at room temperature in the dark for 1 h. Finally, the cytoskeleton of the cells was stained with phalloidin-iFluor 647 (1:1000, ab176759) in 1% BSA for 1 h, and nuclei were stained with DAPI (R37606, Invitrogen). Samples were observed under a scanning laser confocal microscope.

### Statistical Analysis

No statistical tests were used to predetermine the sample size. All data are included for analysis and are presented as the means ± standard deviations (SD). Statistical analysis was performed using GraphPad Prism 10 software. For parametric comparisons, two-tailed Student’s *t*-test was implemented with a *p*-value less than 0.05 being considered a significant difference. All experiments were repeated in triplicate unless otherwise mentioned.

## Supplementary Material



## Notes

Microfabrication of elastomers, initial stress–strain tensile curves, effects of the cycle numbers of freeze–thawing treatment on tensile properties, mechanical testing by compression, small-angle X-ray scattering tests, effects of solvents on the transparency, photographs of PVA hydrogels, stability of STHEs under prolong culture conditions, effects of the incorporation of PAm on tensile properties, standard curve of 5-FU, biocompatibility of STHEs, STHE microwells with different bottoms for tumor spheroid generation, optimization of cell seeding density for tumor spheroid growth, fabrication of mouse tumor tissue-derived spheroids, cell viability of mouse tumor tissue-derived spheroids, characterization of cell line-derived tumor spheroids, cell viability of cell line-derived tumor spheroids, and comparison of STHEs with other common polymeric materials (PDF)
