# Supplementary material for: Highly Strong and Transparent Hydrogel Elastomers Microfabricated for 3D Microphysiological Systems
Source: ACS Appl Mater Interfaces. 2025 Jul 10;17(29):42394–406. doi: 10.1021/acsami.5c07880 (PMC12291088; doi:10.1021/acsami.5c07880)
Supplement: Supplementary file 1 [file am5c07880_si_001.pdf]

Supporting Information

Highly strong and transparent hydrogel elastomers microfabricated for 3D microphysiological systems

Wenxiu Li[1,2,#], Lianxin Li[3,#], Huimin He[1,2], Wang Peng[2], Zhengdong Zhou[2], Wanqing Wu[2], Dong Lv[2], Yaqing Chen[4], Wending Pan[4], Xiaoyu Zhou[2,3], Jun Yin[1]*, and Mengsu Yang[2,3]**

[1]Institute for Frontier Science, Nanjing University of Aeronautics and Astronautics, Nanjing 210016, P. R. China

[2]Department of Biomedical Sciences, and Tung Biomedical Sciences Centre, City University of Hong Kong, Hong Kong SAR, 999077, P. R. China

[3]Department of Precision Diagnostic and Therapeutic Technology, City University of Hong Kong Matter Science Research Institute (Futian), Shenzhen, 518057, P. R. China

[4]Department of Mechanical Engineering, The University of Hong Kong, Hong Kong SAR, 999077, P. R. China

These authors contributed equally to this work: Wenxiu Li and Lianxin Li

*E-mail: hehuimin@nuaa.edu.cn; yinjun@nuaa.edu.cn; bhmyang@cityu.edu.hk

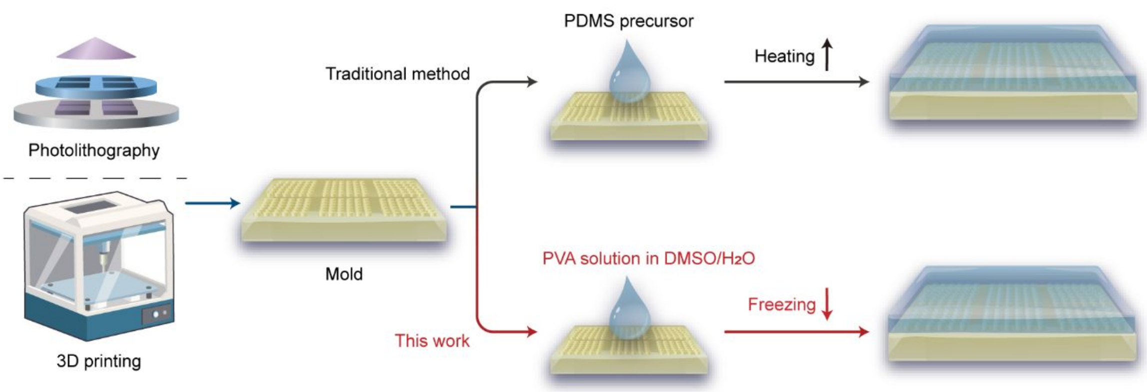

Figure S1. Microfabrication of elastomers. Schematics of the microfabrication of elastomers associated with PDMS and STHEs.

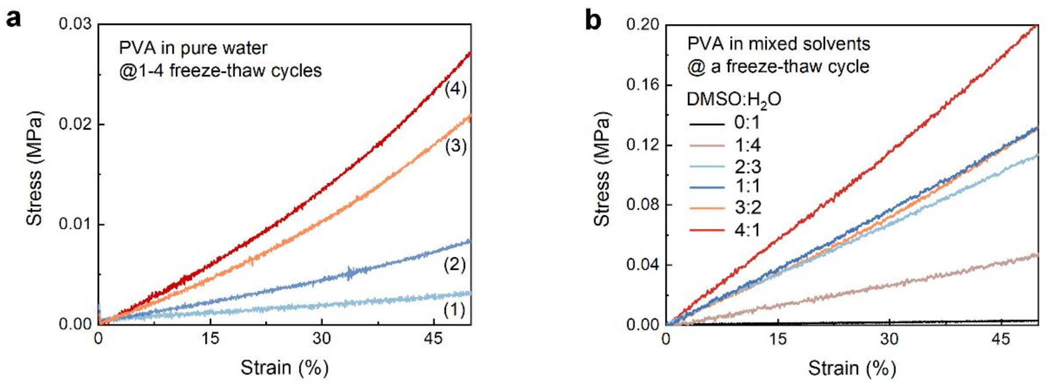

Figure S2. Initial stress-strain tensile curves for PVA hydrogels. a, Tensile responses of PVA hydrogels fabricated from PVA dissolved in pure water with different cycle numbers of freeze-thaw treatment. **b,** Tensile responses of PVA hydrogels fabricated from PVA dissolved in mixed solvents with one freeze-thaw cycle.

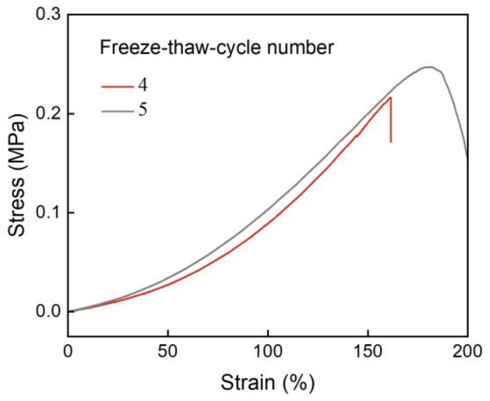

Figure S3. Effects of the cycle numbers of freeze-thawing treatment on tensile properties. Tensile responses of PVA hydrogels fabricated from PVA dissolved in pure water with 4 and 5 cycles of freeze-thaw treatment.

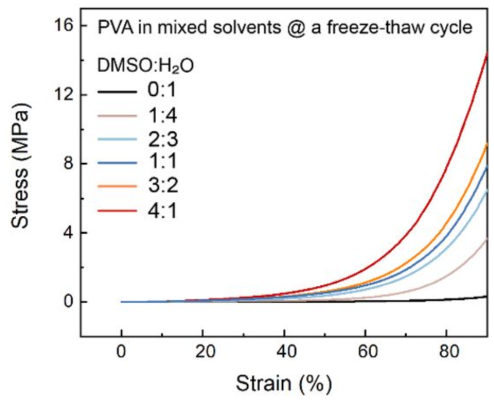

Figure S4. **Mechanical properties of PVA hydrogels tested by compression.** Compressive stress-strain curves of PVA hydrogels.

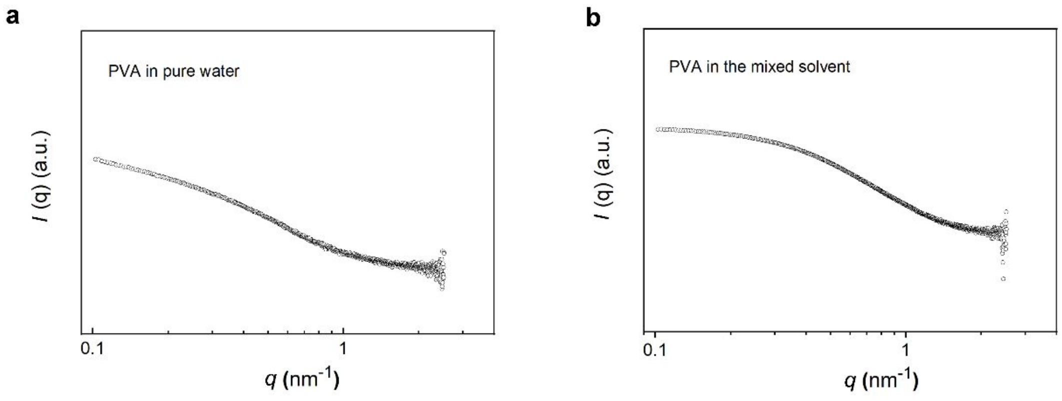

Figure S5. **Small angel X-ray scattering tests for PVA hydrogels. a, b** Scattering intensity (*I*) recorded as a function of scattering vector (*q*) for PVA hydrogels fabricated from PVA dissolved in pure water (**a**) and the mixed solvent (**b**).

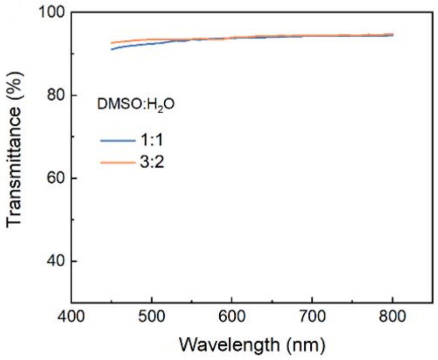

Figure S6. Effects of solvents on the transparency of PVA hydrogels. Transmittance of PVA hydrogels (1 mm thickness) prepared from PVA dissolved in solvents with DMSO and H_2O at weight ratio of 1:1 and 3:2, respectively.

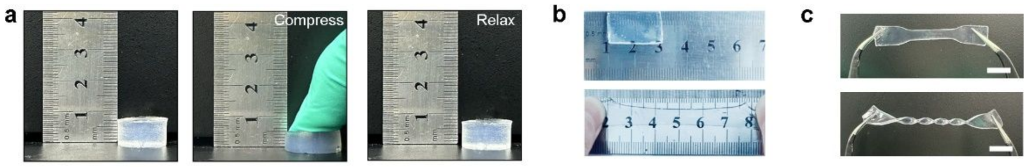

Figure S7. Photographs of PVA hydrogels. a, Photographs of a PVA hydrogel upon force loading and relax. **b, c** Photographs of PVA hydrogels withstanding tension and twisting.

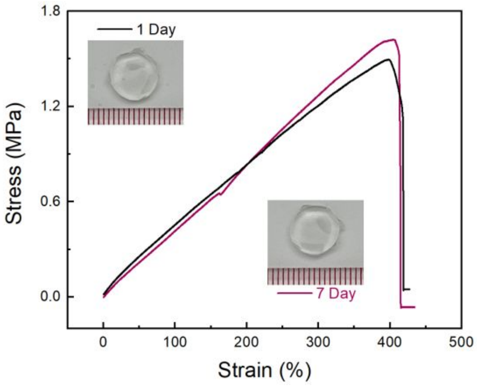

Figure S8. **Effect of prolong culture conditions on the mechanical properties and transparency of STHEs.** Mechanical responses of STHEs after immersion in PBS for 1 day and 7 days respectively (Insets show photographs of STHEs accordingly).

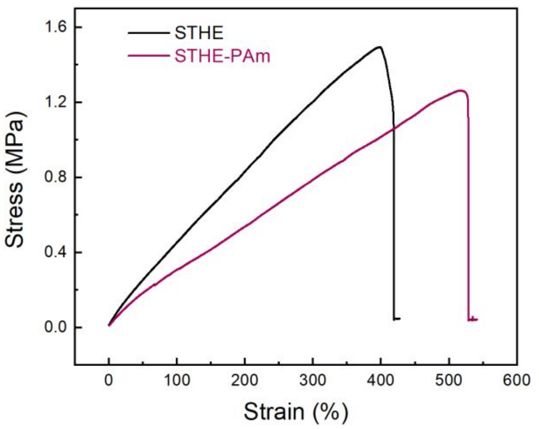

Figure S9. Effects of the incorporation of PAm on tensile properties. Tensile responses of STHE and STHE-PAm respectively.

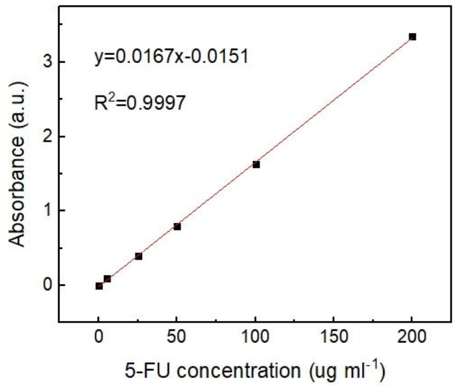

Figure S10. Standard curve to determine 5-FU concentrations. The correlation between the concentration of 5-FU drug (x) and the absorbance of UV light at 266 nm (y).

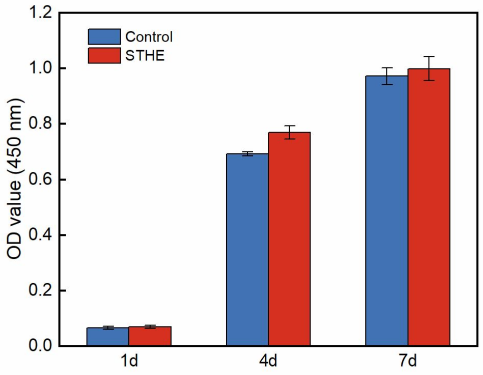

Figure S11. **Biocompatibility of STHEs.** Cell viability of human umbilical vein endothelial cells (HUVECs) cultured with and without STHE-conditioned medium, as determined by Cell Counting Kit-8 (CCK-8) assay. No significant difference in cell viability was observed between the control and STHE groups.

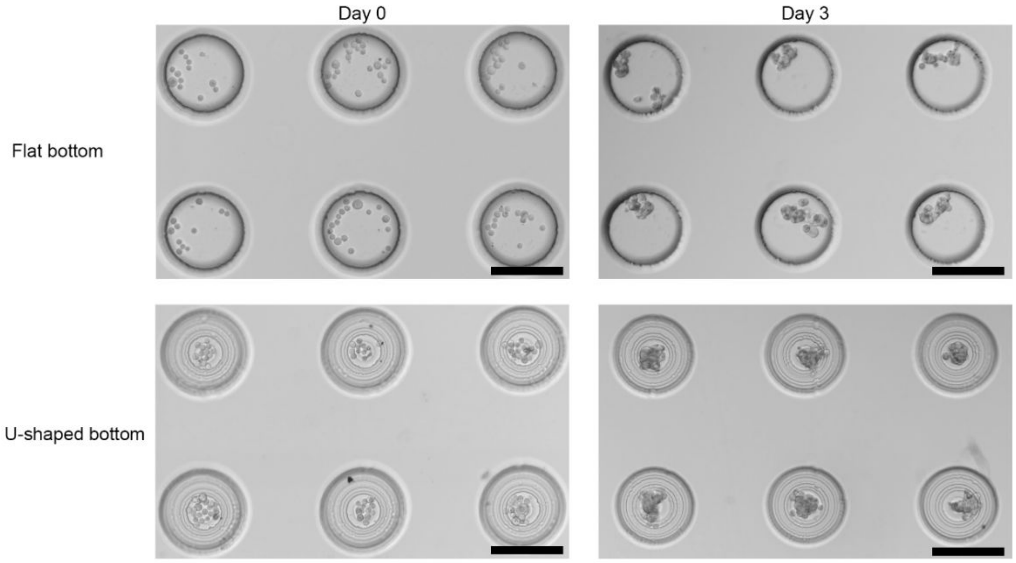

Figure S12. STHE microwell arrays with flat or U-shaped bottom for tumor spheroid generation. The bright fields of A549 cells cultured in flat-bottom or U-shaped STHE microwell arrays on day 0 and day 3, respectively. Scale bar: 200 μm.

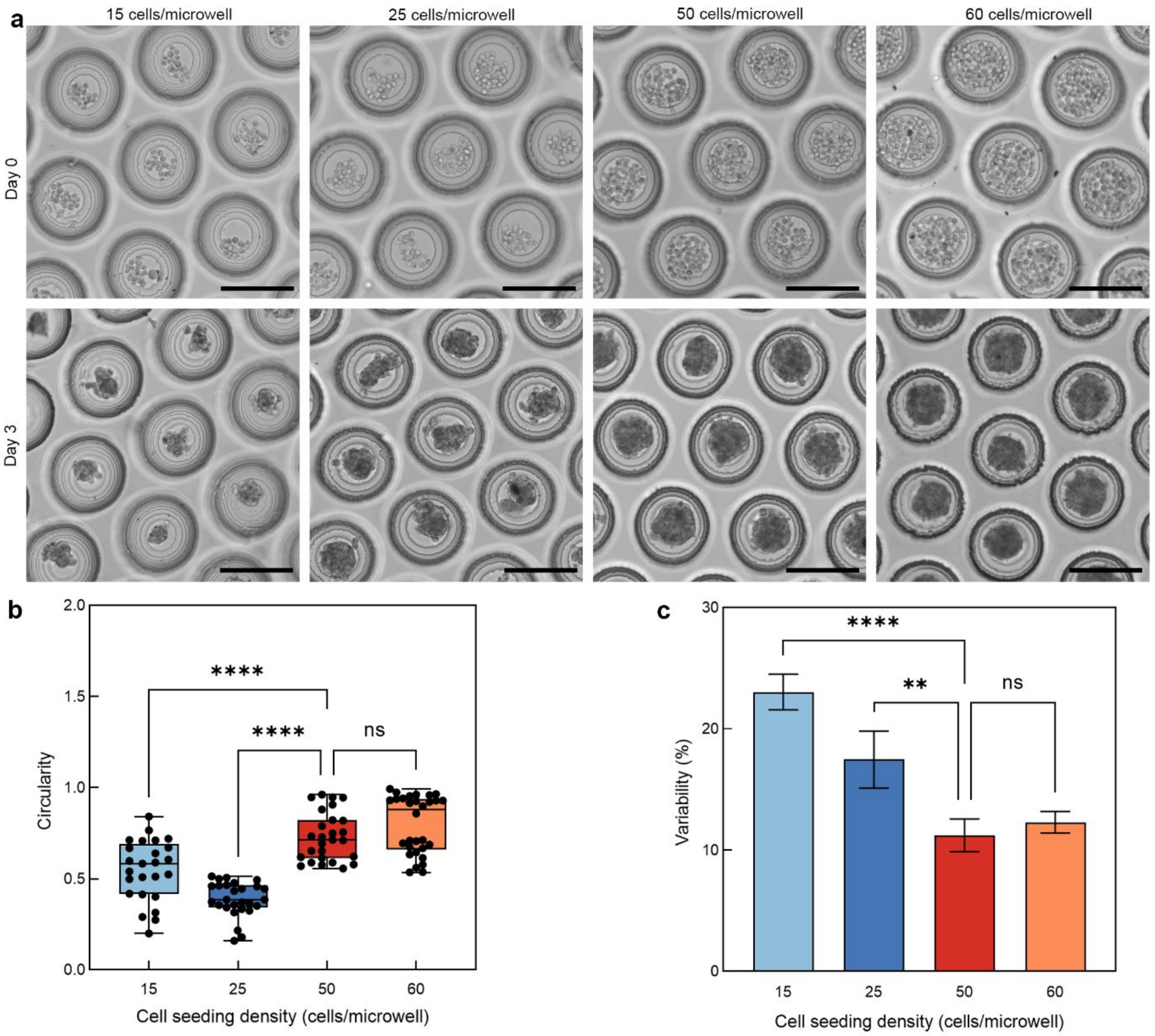

Figure S13. **Optimization of cell seeding density for tumor spheroid growth in STHE microwells. a,** Brightfield images of A549 cells cultured in STHE microwells with different initially seeded cell densities per microwell at different culture time. Scale bar: 200 µm. **b, c** Quantified results of spheroid circularity (**b**) and size variability (**c**) after 3 days of culture.

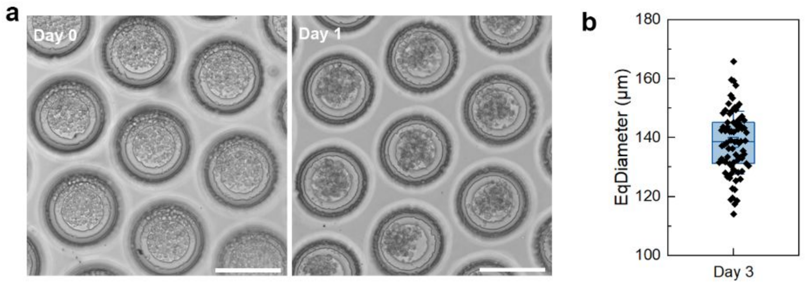

Figure S14. **Fabrcation of mouse tumor tissue-derived spheroids. a,** Brightfield images of mouse tumor cells cultured in U-shaped microwell arrays at different culture time. Scale bar: 200 µm. **b,** Quantified results of tumor spheroid sizes after 3 days of culture.

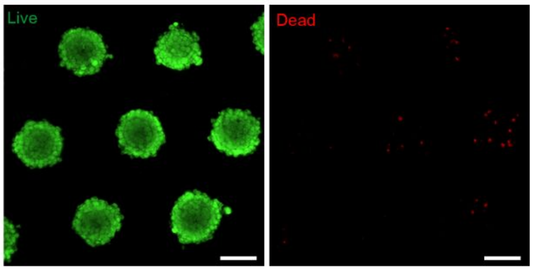

Figure S15. **Characterization of cell viability of mouse tumor tissue-derived spheroids by Live/Dead staining.** Live cells stained in green, while dead cells stained in red after 3 days of culture. Scale bar: 100 μm.

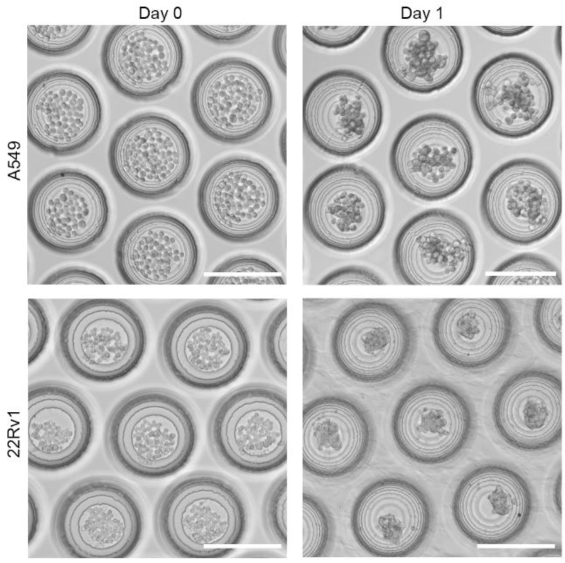

Figure S16. **Characterization of cell line-derived tumor spheroids.** Brightfield images of cell line-derived tumor spheroids growth in U-shaped microwell arrays at different culture time. Scale bar: 200 μm.

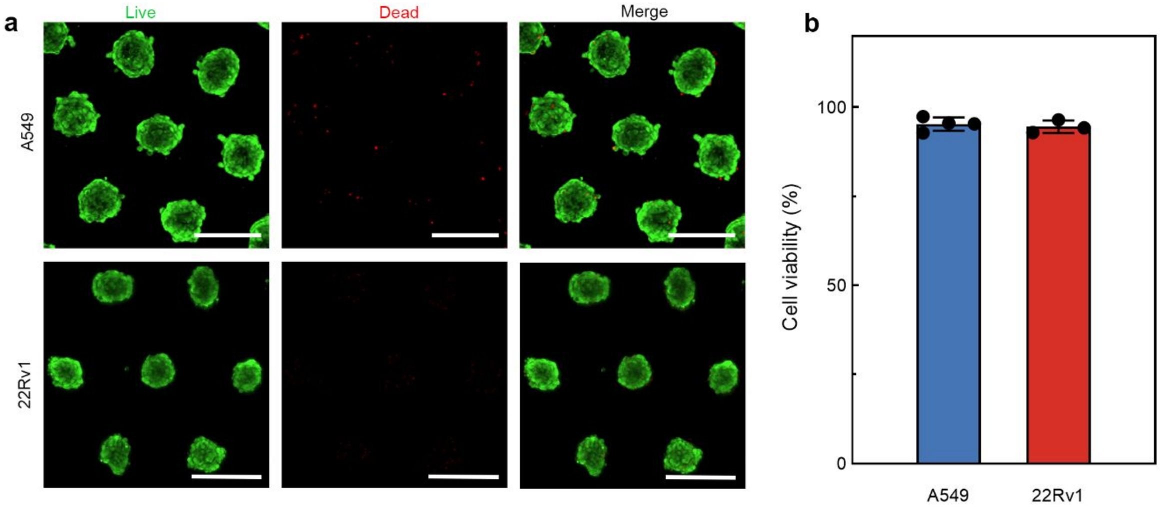

Figure S17. **Characterization of the cell viability of cell line-derived tumor spheroids by Live/Dead staining. a,** Fluorescence images of tumor spheroids stained by Live/Dead staining after 3 days of culture. Live cells were stained in green, while dead cells were stained in red. Scale bar: 200 μm. **b,** Quantification of cell viability of the tumor spheroids.

Table S1 Comparison of STHEs with other common polymeric materials.

	Toughness ($MJ\ m^{-3}$)	Ductility (%)	Transparency (%)	Modulus (MPa)	Strength (MPa)	Ref.
STHE	2.82	362.9	94.77	0.45	1.6	This
PDMS	1.99	153.5	94.91	1.89	2.65	work
PVA	0.12	175.9	90.5	0.042	0.24	
PAm	0.006	110.4	97.47	0.008	0.00749	
Agarose	0.02	12.1	83.74	1.0	0.078	
Gelatin	1.65	220	94	1.25	1.95	1
Chitosan	0.015	53.8	90	0.11	0.05	2
Cartilage	2.7	37.5	15	7	17.5	3
Skin	0.14	40	45	0.4	3	4

Note: PVA denotes the hydrogels prepared from PVA in pure water; The toughness is estimated by integrating the tensile stress-strain curves.

References

[1] S. Sharifi, M. M. Islam, H. Sharifi, R. Islam, D. Koza, F. Reyes-Ortega, D. Alba-Molina, P. H. Nilsson, C. H. Dohlman, T. E. Mollnes, Bioact. Mater. 2021, 6, 3947.
[2] C. Zhao, X. Gong, L. Shen, Y. Wang, C. Zhang, ACS Appl. Polym. Mater. 2022, 4, 4025.
[3] E. Danso, J. Honkanen, S. Saarakkala, R. Korhonen, J. Biomech. 2014, 47, 200.
[4] P. Gasson, R. J. Lapeer, V. Karri, presented at Proceedings of the Internation Conference on Polymers and Moulds Innovations 2009.
